# Restricting range restricts conclusions

**DOI:** 10.3389/fpsyg.2014.00569

**Published:** 2014-06-12

**Authors:** Nemanja Vaci, Bartosz Gula, Merim Bilalić

**Affiliations:** Department of General Psychology and Cognitive Science, Institute of Psychology, Alpen-Adria University KlagenfurtKlagenfurt, Austria

**Keywords:** expertise, skill acquisition, chess, Elo rating, gender differences, gerontology, talent

## The expertise approach and skill acquisition

Research on expertise is by definition focused on a restricted sample of individuals. Experts are people who consistently produce outstanding performance in their domains (Ericsson, 2006) and as such are without exception located on the positive side of the skill distribution. The usual approach in the study of expertise is to compare the extreme group of the skill distribution, experts, with the extreme group at the other end, that of novices. This contrasting approach, which we have called the “expertise approach” (Bilalić et al., 2010, 2012), has a long tradition (Chase and Simon, 1973; Simon and Chase, 1973; De Groot, 1978; Preacher et al., 2005). Its main advantage over the common approach in cognition, where all participants are at the same skill level, is the presence of a control group of novices that enables falsification of results obtained on experts (Wason, 1960; Kuhn, 1970; Campitelli and Speelman, 2013). In that way, the expertise approach is not unlike the neuropsychological approach that contrasts results obtained on patients with the results of “normal” participants (Shallice, 1988).

The main goal of the expertise approach is to provide evidence relating to the cognitive and neural mechanisms behind processes such as object and pattern recognition, which would be difficult to obtain from subjects who possess approximately the same level of expertise. The skill acquisition process, which is one of the main topics of expertise (William and Harter, 1899), is of secondary importance in the expertise approach. This is understandable as the contrast between experts and novices captures only the beginning and the end product of the skill acquisition process. It is unrealistic to follow people for the length of time required in order to achieve expertise in a given domain. However, expertise researchers have recently started employing an archival approach that provides a more complete picture of the skill acquisition process (Charness and Gerchak, 1996; Chabris and Glickman, 2006; Howard, 2008, 2009; Bilalić et al., 2009). In the game of chess, a domain commonly studied in expertise research, there are precise records of all practitioners from an early age (Howard, 2006a; Bilalić et al., 2009). These records include not only personal information such as gender and age, but also skill levels at different stages, numbers of games played, and corresponding skill change. The records provide a wealth of data for investigating the influence of factors such as age, gender, and even talent, on the skill acquisition process. Here we want to draw attention to the fact that some of the databases used in previous research only provide records of the very best practitioners. In the expertise approach, such restriction is an integral part of the methodology, but restricting the range of population in the archival approach could have grave consequences for the conclusions about the nature of skill acquisition.

## Different databases, different conclusions

One of the advantages of chess as a domain is that there is an objective and reliable measure of skill. Skill is measured on an interval scale that reflects the performance of a player against other players. The Elo rating, named after Arpad Elo who introduced the scale as a measure of chess skill (Elo, 1978), is measured in the same way all over the world. A beginner is supposed to have 600–800 Elo points, average players about 1500 Elo points, master players above 2200 Elo points, while the very best players, called grandmasters, have ratings above 2500 Elo points. Expert players are considered to have above 2000 rating points.

The most frequently used database in skill acquisition studies is the database of the International Chess Federation, FIDE (for more information, see Howard, 2006a). This database, like other chess databases we will mention here, offers multiple advantages for skill acquisition research. Firstly, it gathers records from the 1970s to the present, and so it is possible to obtain trajectories of ratings over the course of players' lives. Secondly, it represents the whole population of the very best players in the world. Thirdly, this database contains multiple measurement points from players, so it can be used to observe individual skill trajectories. Besides rating points, numerous other variables are recorded (e.g., number of games played, gender of participants, nationality, title, rating change, rating rank) which could be used for research purposes (Howard, 2006a). In other words, the FIDE database offers a fruitful basis for exploration and description of the multiple factors and processes behind chess skill acquisition.

For all its advantages, the FIDE database provides only the records of the very best players. Due to technical and logistical reasons, the FIDE database at the beginning logged only master level players (above 2200 Elo). Only in the 1990s was the level lowered to expert level players (2000 Elo) and then in the last decade to the level of average players (1500 Elo and below). In other words, the worst players in the FIDE database are still average practitioners.

The ideal situation would be to have records of all players from the very beginning of their careers, not only when they reach a particular level of expertise. In this scenario, the database would also encompass people who for whatever reasons do not become experts. Fortunately, there are such databases. National databases, such as the databases of the German Chess Federation and the United States Chess Federation (USCF), keep records of all their members and thus represent the whole population of competitive (national) players. They provide all the information the FIDE database offers without restricting the range of skill.

If one is trying to examine factors that influence skill acquisition, a database that contains only average and above-average players should not be the starting point of investigations. As is well known in the field of statistics, the conclusions obtained on data with restricted range could be misleading especially if they are not obtained by appropriate analyses (Long, 1997; Sackett and Yang, 2000). The best possible way to examine differences in effects that researchers could obtain while analyzing the data is through the comparison of effects made on different databases. Here we illustrate the possible pitfalls of skill range restriction by comparing distributions of ratings from a database with restricted range (FIDE) and a database with unrestricted range (German).

**Figure 1:**
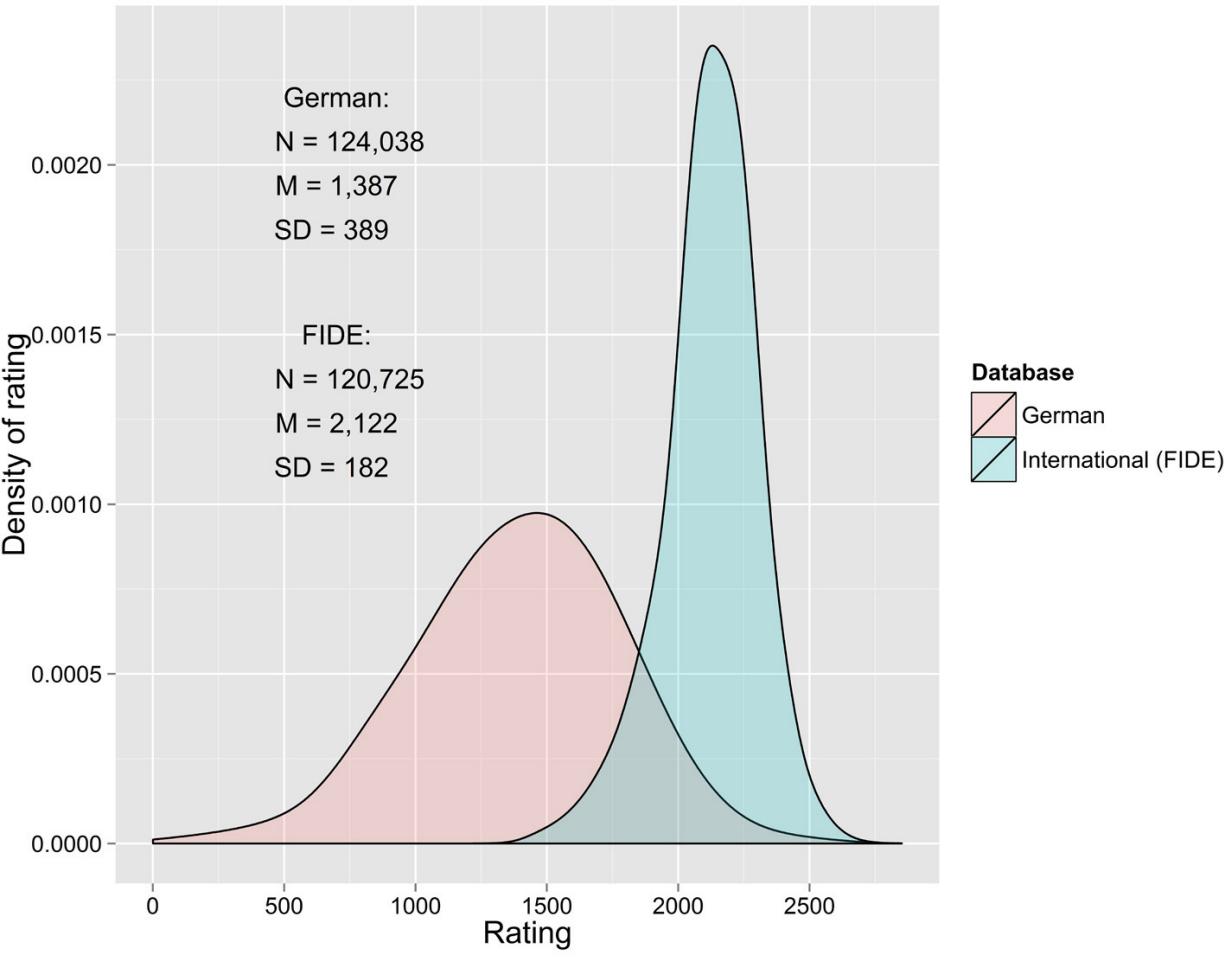
**Distribution of chess skill as measured by Elo rating in FIDE (blue color) and German (red) databases (as of 2008)**. The databases contain similar number of players, but differ vastly in the distribution shape and coverage—the only overlap is at the highest values of the German database and lowest values of the FIDE database.

The FIDE and German databases contain similar number of practitioners, around 120,000 (see Figure 1). However, the ratings of the two distributions overlap only at the highest values of the German distribution and the lowest values of FIDE distribution. Not only are the mean and variance of both distribution vastly different, but also other parts of the distribution, such as quartiles, are also extremely different.

**Figure 2:**
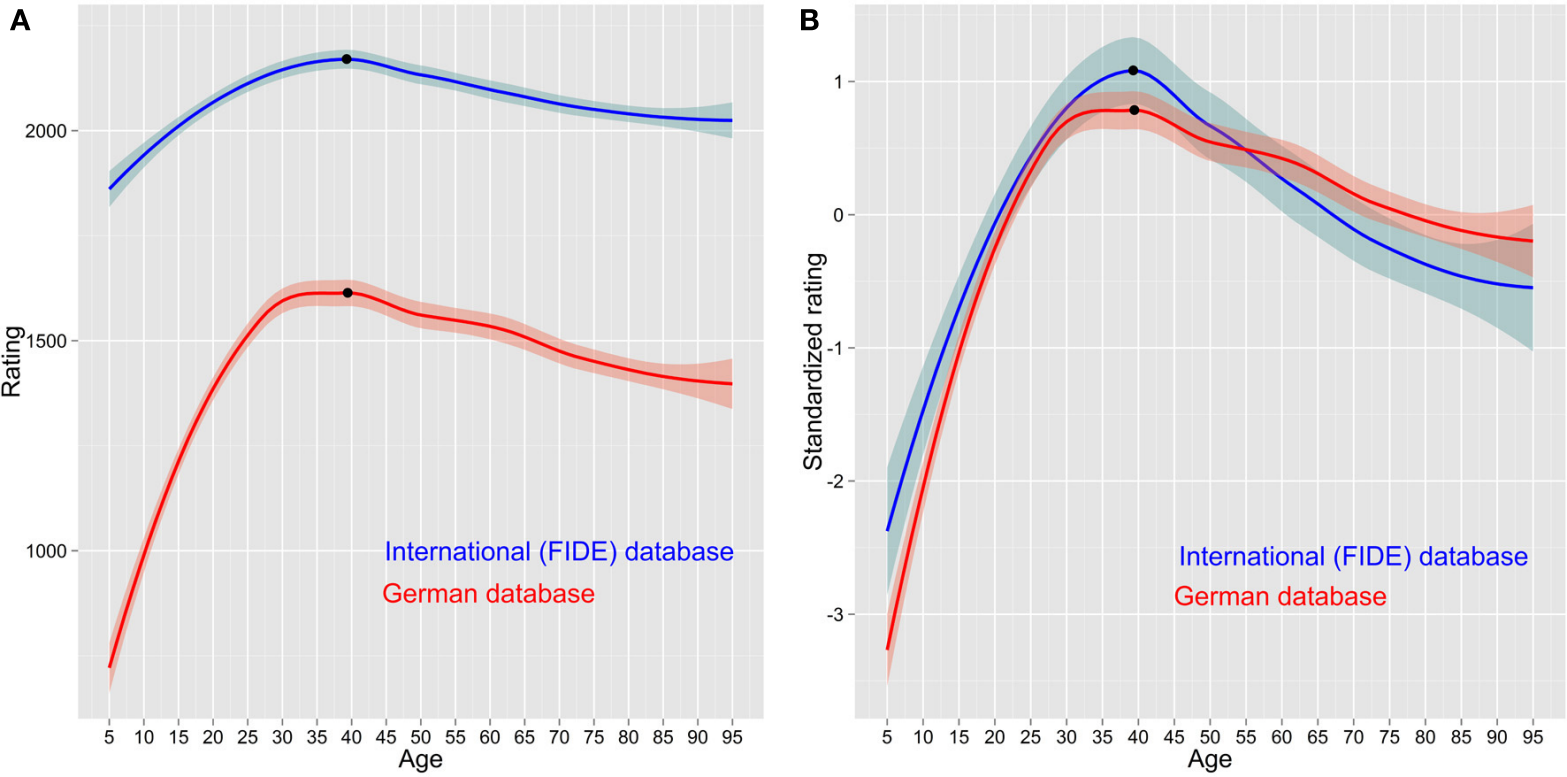
**(A)** Average skill trajectories, in Elo with 95% confidence intervals, over the years in FIDE database (blue color) and German (red) database. **(B)** Average standardized skill trajectories, in *Z*-values with 95% confidence intervals, over the years in FIDE database (blue color) and German (red) database.

Restricting the database to the best players also has a consequence for the skill trajectories of players. One needs time to become an expert and the players in the FIDE database are older (37 years) than the players in the German database (32 years). The differences between the two databases are also evident when we compare typical skill trajectories. Figure 2A shows the FIDE players entering the database at around age 10, having already become competent players (rating of 1900 Elo), with a subsequent shallow increase to the peak at age 39. In contrast, the German players have a steeper increase, since they are entered the database as novices and learn faster at beginning skill level, as implied by the power law of practice (Newell and Rosenbloom, 1981), until the same peak at age 39. The decline in later years is also different in the two databases with FIDE players declining faster than their German counterparts.

One could say that the FIDE and German players have vastly different ratings that make the comparison between them difficult. One way around this problem is to standardize ratings in each database separately and check if the skill trajectories are similar in both datasets. Figure 2B shows the standardized rating as a function of age. Again, on average FIDE players start at a higher skill level but improve more slowly. They also have a higher peak but their decline is so rapid that in the latter stages of their careers their standardized performance is lower than the standardized performance of their German colleagues.

## Explaining contradictory results

We have demonstrated that there are vast differences between the databases commonly used in the archival approach to skill acquisition. The two datasets are completely different in the range of values as well as in the number of participants that are obtaining a particular rating. The restricted databases, such as FIDE, do not represent the whole skill range and may provide inadequate answers to the questions under investigation. The restriction of range and its consequences may also explain some of the inconsistencies and contradictory findings in the field.

For example, Roring and Charness (2007) used the FIDE databases to investigate age effects on skill acquisition. They demonstrated the peak age in chess skill to be around 43 years, much later than previously proposed peak around 35 years (e.g., Howard, 2005). Another surprising result was the fact that the decline is steeper for initially lower rated participants than for higher rated participants. In other words, initially more able participants were declining significantly more slowly than their initially weaker colleagues. Our illustrations (Figures 2A,B) indicate that both peak age and declining rate are influenced by the range restriction in the FIDE database. The conclusion would be significantly different if a whole range database, such as the German database, were used.

To further illustrate possible consequences of the range restriction, we can consider the inconsistent findings in the research on gender differences in skill acquisition. It is notable that the studies using the restricted FIDE database regularly find gender differences in skill acquisition (Howard, 2005, 2006b, 2014 but see, Bilalić and McLeod, 2006, 2007). Furthermore, the studies using the national German and USCF databases (Chabris and Glickman, 2006; Bilalić et al., 2009) also noted the differences in the mean and highest ratings of women and men. However, using the unrestricted range and the full lifespan data, they observed that factors such as participation rates and dropout rates could explain the differences. This kind of analysis is impossible with the FIDE database where dropouts are not recorded because the people concerned stopped playing chess before they achieved expert level.

Researchers using the FIDE database to investigate talent (Howard, 2008, 2009) face a similar problem. The time required to reach a certain level and the amount of practice (as measured by games played) may well provide clues about the different natural endowments of certain players. This in turn may allow us to speculate about different levels of talent. It is, however, impossible to make any certain conclusions if we lack the very first part of their skill acquisition process, as we do in the FIDE database. As with the gender factor in skill acquisition process, the differences in the early stages may as well overshadow the differences at the highest level. Similarly, the causes behind dropouts may remain unresolved because the data of the people who for whatever reasons stopped playing chess is not available.

Both the expertise and archival approaches are important vehicles for the investigation of expertise and cognition in general. The restricted range of focus in the expertise approach is a fundamental part of the methodology and an advantage over usual research on cognition. The archival approach offers the possibility of capturing the full cycle of the long-term skill acquisition process. In this paper we have demonstrated that the results obtained on the restricted range do not necessarily generalize to the whole range of values. The effects obtained with restricted range cannot and should not be used to make inferences about the mechanisms and factors that influence skill acquisition. When we restrict our data, we restrict our conclusions too.

### Conflict of interest statement

The authors declare that the research was conducted in the absence of any commercial or financial relationships that could be construed as a potential conflict of interest.
